# Behavioral Correlates of Empirically-Derived Dietary Patterns among University Students

**DOI:** 10.3390/nu10060716

**Published:** 2018-06-03

**Authors:** Megan P. Mueller, Stacy A. Blondin, Ariella R. Korn, Peter J. Bakun, Katherine L. Tucker, Christina D. Economos

**Affiliations:** 1 Department of Community Health Sciences, Fielding School of Public Health, University of California-Los Angeles, Los Angeles, CA 90095, USA; 2 Harvard T.H. Chan School of Public Health, Harvard University, Boston, MA 02115, USA; sblondin@hsph.harvard.edu; 3 Friedman School of Nutrition Science and Policy, Tufts University, Boston, MA 02111, USA; ariella.korn@tufts.edu (A.R.K.); peter.bakun@tufts.edu (P.J.B.); christina.economos@tufts.edu (C.D.E.); 4 Clinical Laboratory and Nutritional Sciences, Center for Population Health & Health Disparities, University of Massachusetts-Lowell, Lowell, MA 01854, USA; katherine_tucker@uml.edu

**Keywords:** university students, dietary patterns, health behaviors, emerging adulthood

## Abstract

Given the importance of young adulthood in establishing lifelong dietary habits, it is imperative to better understand potential underlying drivers of dietary behavior in the university-age population. Dietary patterns have been associated with disease risk, but behavioral predictors of dietary pattern adherence are poorly understood, especially among emerging adults. This study aims to evaluate health-related behaviors associated with dietary pattern scores among freshmen participating in the Tufts Longitudinal Health Study (TLHS; *n* = 630). We previously derived dietary patterns using principal components analysis and orthogonal rotation from dietary intake data. Health-related behavior data were collected via survey. All data were collected during the Spring semesters of 1998–2007. Unadjusted linear models were used to determine associations between dietary pattern scores and health-related behaviors. Significant correlates were retained in multivariable regression models, which were adjusted for demographic characteristics. We found that never eating meals away from home was associated with higher adherence to the Prudent and lower adherence to the Western and Alcohol patterns. Intention to lose weight was negatively associated with the Western pattern, while intention to gain weight was positively associated with all dietary patterns. These findings suggest that intervention efforts aimed at improving eating out behaviors and engaging in healthy weight management strategies may promote healthier dietary patterns among university students.

## 1. Introduction

Facing more than 200 food-related decisions daily [1], dietary choices are among the most frequent of human behaviors [2]. Diverse, complex, and interacting factors at the individual, interpersonal, environmental, and policy levels [3] influence an individual’s pattern of eating over time [2,4,5,6]. Dietary patterns, in turn, influence chronic disease risk and quality of life [7,8,9]. Therefore, determining which factors influence dietary patterns across the lifespan could inform interventions to improve diet quality and associated health outcomes. Food choice is also subject to changes over the life span [6] and is person-, time-, and context-specific [10,11].

The transition between adolescence and adulthood, characterized by increasing independence, autonomy, and responsibility, is often the first time period during which young adults make their own decisions about “how, what, where, and when to eat” [12]. Moreover, there is evidence that weight gain during early and middle adulthood is significantly associated with chronic disease risk later in life [13]. Therefore, early adulthood is a crucial life-stage for establishing life-long health behaviors and habits, including healthy eating patterns [14].

For many, the transition into adulthood results in a shift in composition and quality of the diet, with most studies suggesting a worsening of diet quality during this transition [12,14]. Data from the National Health and Nutrition Examination Survey (NHANES) suggest that people aged 12–39 years consume the greatest percentage of total energy from sugar-sweetened beverages per day, compared to younger and older age groups [15,16]. This age group is also the most likely to fall short of meeting fruit and vegetable serving recommendations [17]. Longitudinal studies support these findings, showing that consumption of fruit, vegetables, and milk generally decreases during the transition to early adulthood, while intake of sweetened beverages and snack foods tends to increase [18,19,20]. The transition to young adulthood has also been associated with increased frequency of fast food consumption [21]. Yet, few studies have examined correlates of dietary patterns among university students.

We know of only one previous study in the US that evaluated predictors of empirically defined dietary patterns among young adults. In a prospective analysis of the Project EAT cohort, socioeconomic status, family meal frequency, and home availability of healthy food were positively associated with vegetable and fruit, and starchy, food patterns and inversely associated with a fast food pattern among 18–21 year olds [22]. In contrast, home availability of unhealthy food was inversely associated with the vegetable and fruit and starchy food patterns and positively associated with the fast food and snack food patterns. Parental and peer support for healthy eating were positively associated with the vegetable and fruit pattern and inversely associated with the fast food pattern. While the Project EAT study contributes to the understanding of behavioral factors related to dietary patterns, this analysis grouped high school- and university-age individuals together and did not explicitly evaluate behavioral factors specific to the lived experiences of many university students—many of whom live independently of their families and whose dietary patterns may be more impacted by individual-level factors and/or their peers [23].

Given the deficit of literature examining associations between underlying health behaviors and adherence to dietary patterns among university students, the aim of this analysis was to investigate cross-sectional associations between sociodemographic and health-related behaviors and dietary patterns among undergraduate students in the northeastern United States. Identifying factors that influence students’ dietary patterns could inform policies, programs, and interventions in the university setting, to improve diet quality and, thereby, reduce lifelong chronic disease risk of current and future generations.

## 2. Materials and Methods

### 2.1. Sample and Procedures

**Figure 1:**
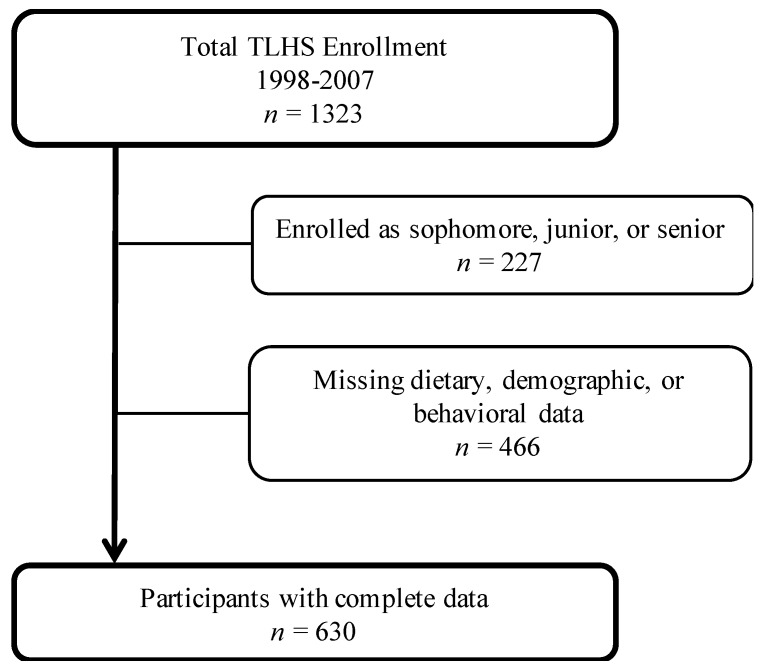
Flow chart depicting the final analytic sample. Boxes to the right indicate the number of participants excluded for each reason.

As a part of the Tufts Longitudinal Health Study (TLHS), dietary and behavioral data were collected annually between 1998–2007 from participating Tufts University freshmen using the Block Food Frequency Questionnaire (FFQ) and a 40-item Health Behavior Survey (HBS). Enrolled freshman completed an initial HBS in the summer prior to the start of their first year. All participating students were also invited to attend follow-up health assessments in the Spring semester, where they completed a HBS, a FFQ, and direct anthropometric measures. For this analysis, the sample was restricted to freshmen with complete behavioral, demographic, dietary, and physical activity data obtained during the Spring assessment (*n* = 630; See Figure 1). This group of freshmen students maximized the number of participants, enabling us to better compare these findings with previously published work [22]. This age group also allowed us to capture potential behavioral factors of importance during an especially intensive transition period from high school to university [12,14,24].

### 2.2. Measures

A description of the FFQ measure and dietary pattern scoring/determination was published previously [24]. Briefly, participants were asked to report how frequently they consumed each of the food items listed in the following food categories: Fruits/juices, breakfast, vegetables, meat/fish/poultry, breads/snacks/spreads, dairy, sweets, and beverages. FFQ data were processed via the University of Minnesota Nutrition Data System for Research (NDSR) and the Epidemiology and Dietary Assessment Research Program at the US Department of Agriculture Human Nutrition Research Center on Aging at Tufts University [25]. The FFQ food items (~100) were further condensed into 43 food groups, adapted from Hu et al. (2000) [26]. We used results from a previous principal component analysis (PCA), in which dietary patterns in the TLHS study were identified [24]. Three dietary patterns were derived from the PCA based on scree plots and eigenvalues and labeled based on factor loadings and previous literature. The scree plot used to identify the variability captured by each factor is presented in Supplementary Figure S1 from Blondin et al. 2015 [24]. The three factors with eigenvalues >2 were retained and then orthogonally rotated using varimax rotation. In total, these three dietary patterns explained 24.4% of the variance in dietary intake. Food groups with factor loadings ≥3 in absolute value were used to interpret the factors. Each participant received a score for each dietary pattern, which was calculated by summing the standardized values for the food items and weighting by the relative factor loadings.

Behavioral factors of interest were identified based on previous literature evaluating associations between each factor and diet quality [12,14,27,28,29,30,31,32,33,34,35,36,37,38,39,40,41,42]. All behavioral factors were assessed using the spring semester follow-up HBS, which was developed using existing population-based surveys and piloted with over 100 students between 1998 and 1999, as described in Blondin et al. (2015) [24]. To determine frequency of dining at the dining hall, dining at restaurants/takeout (referred to herein as “eating out”), or preparing meals at home, participants were asked to identify how they obtained most of their meals in the last year on a four-point scale (never, rarely, sometimes, or often). Participants also indicated whether they lived on or off campus during the academic year. To determine television use, participants were asked to record how many hours a day they spent watching television (0, less than 1 h, 1, 2, 3, 4, or 5 or more hours). Getting adequate sleep was defined as the participant indicating they agree or strongly agree to the statement “I usually sleep at least 7–8 h every night”.

Perceived influence of friends, family, “religion/culture”, living situation, food costs, nutrition, and food availability on food choice was evaluated via a five-point Likert scale (strongly positive, positive, no effect, negative, strongly negative). Positive and negative responses were combined into single response categories (strongly positive/positive and strongly negative/negative). Intention to lose or gain weight was determined by the participant affirming that they had intentionally tried to lose or gain weight in the past year. Perceived life control was assessed using a ten-point scale (1 indicating that the participant felt they were not in control at all, and 10 indicating that the participant felt they were completely in control). Smoking status was determined by whether the participant indicated that they were a current smoker (yes/no).

Methods for calculating total Metabolic Equivalent of Task (MET) minutes of physical activity were published previously [24]. In brief, participants reported how often and how long they engaged in physical activity and exercise over the past three months for activities engaged in at least once a week using the Cooper Institute for Aerobics Research Aerobics Center Longitudinal Study (ACLS) questionnaire [43,44,45]. MET minutes were calculated by multiplying the number of sessions by the duration of each session. For endurance activities, intensity was determined by dividing the distance per session by the duration of each session. When exact values were not available in the MET compendium, we used the MET value or average of MET values of activities that were most closely related to the ACLS activity. Being physically active was defined by ≥500 MET minutes/week.

Demographic factors (age, gender, and race) were also determined via the HBS. Data on socioeconomic status (SES) was not collected. BMI was calculated using direct weight (kg) and height (m^2^) measurements from the Spring assessment. All participants were measured in light clothing without shoes. Weight was measured on a portable balance beam scale (Healthometer, Boca Raton, FL, USA) and recorded to the nearest quarter pound. Height was measured without shoes using a portable stadiometer (Model 214, Seca Weighing and Measuring Systems, Hanover, MD, USA) and recorded to the nearest 1/8 inch.

### 2.3. Data Analysis

All descriptive and exploratory analyses were conducted using SAS Version 9.4 (Cary, NC, USA). Bivariate associations between behavioral factors and dietary pattern scores were evaluated for: frequency of eating in the dining hall, eating out, or preparing meals at home; perceived influence of friends, family, religion/culture, living situation, nutrition, food costs, and food availability on food choices; whether participants lived on or off campus; television use; getting adequate sleep; intention to lose or gain weight in the past year; perceived life control; MET minutes of physical activity; and smoking status. Behavioral factors that were significantly associated with any of the dietary patterns were included in the final models (Supplementary Table S1).

Relationships between predictor variables and dietary pattern score were determined using multivariable regression models, adjusted for age, gender, and race. Effect modification by gender and race/ethnicity was tested for all behavioral factors, as previous research has found differences in dietary patterns and diet-related behaviors between males and females and between racial/ethnic groups [35,46,47,48,49,50,51,52]. For these sub-analyses, race/ethnicity was recategorized as Non-Hispanic white and all other races/ethnicities (including African Americans, Hispanic/Latino, Asian Pacific Islander, American Indian and Alaskan Native, and multi-racial), as less than 25% of our sample identified as other than non-Hispanic white.

## 3. Results

### 3.1. Sample Characteristics

The analytic sample included 630 freshmen with complete behavioral, demographic, dietary, and physical activity data (Figure 1). Participants were predominantly non-Hispanic white (75.4%), and the majority (65.9%) were female (Table 1). The mean age was 18.5 y (SD = 0.5 y). Participants were generally within the range of healthy weight (mean BMI: 22.8 kg/m^2^; SD = 3.0 kg/m^2^), predominantly non-smoking (97.5%), and physically active (83.8%). All participants lived on campus, and the majority reported eating in the dining hall *sometimes or often* (99.7%).

### 3.2. Dietary Patterns

**Figure 2:**
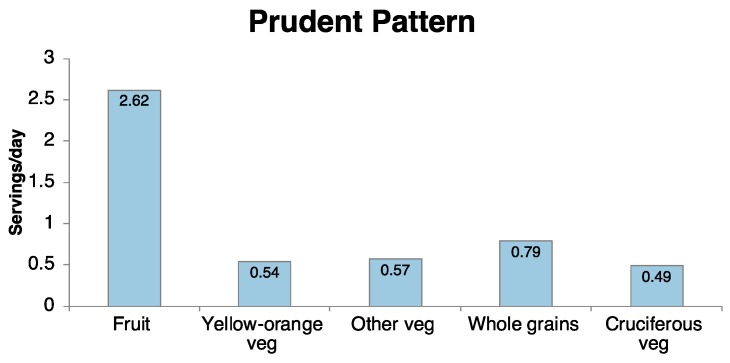

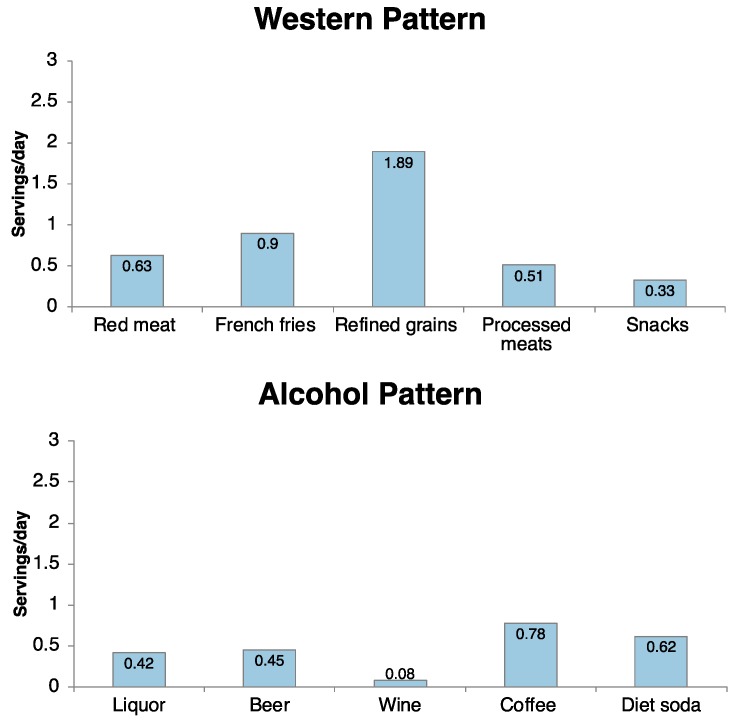
Mean daily medium serving intakes of participants with scores in the top quartile of the three dietary patterns for the foods/food groups with the highest factor loadings. Medium serving sizes of food items are as follows: Red meat (4.2 ounces), French fries (0.75 cups), Refined grains (1 piece), Processed meats (2 pieces), Snacks (1 cup), Fruit (0.5 cups), Dark yellow-orange vegetables (0.5 cups), Other vegetables (0.5 cups), Whole grains (0.67 cups), Cruciferous vegetables (0.5 cups), Liquor (1.5 fl oz), Beer (12 fl oz.), Wine (5 fl oz), Coffee (6 fl oz), Diet soda (12 fl oz).

Three dietary patterns were derived from the PCA: Prudent, Western, and Alcohol (Figure 2) [24]. The Prudent dietary pattern was characterized by daily consumption of fruit (2.6 servings), yellow-orange, cruciferous, and other vegetables (0.5, 0.5, and 0.6 servings, respectively), and whole grains (0.8 servings). The Western pattern was characterized by refined grains (1.9 daily servings), French fries (0.9 servings), red and processed meats (0.6 and 0.5 servings, respectively), and snacks (0.3 servings). The Alcohol pattern included consumption of liquor, beer, and wine (0.4, 0.5, and 0.1 daily servings), in addition to coffee (0.8 servings) and low-energy drinks (referred to herein as diet soda, 0.6 servings).

### 3.3. Behavioral Factors and Dietary Patterns

In multivariable models adjusted for age, race, and gender, *never* eating out (compared to *frequently*) was significantly associated with adherence to the Prudent dietary pattern (*β =* 0.54, *p* = 0.037). Eating out sometimes, rarely, or never was associated with lower adherence to the Western dietary pattern compared to eating out frequently (*β =* −0.91, −0.69, and −0.63, respectively; *p* ≤ 0.001 for all coefficients). Among all participants, eating out *never* or *rarely* was associated with lower adherence to the Alcohol dietary patterns compared to eating out *frequently* (*β =* −0.85, *p* = 0.001 and *β =* −0.54, *p* = 0.009, respectively; Table 2). Those who reported that their family members had a *negative* or *strongly negative* influence on their own food choices (compared to *no effect*) had lower adherence to the Western pattern (*β =* −0.33, *p* = 0.021), while having a *negative or strongly negative* influence on food choices from friends was associated with greater adherence to the Alcohol pattern (*β =* 0.26 , *p* = 0.017). Participants who reported that nutrition had either a *negative* or *strongly negative* or *positive* or *strongly positive* influences on their food choices had higher adherence to the Prudent pattern than those who reported that nutrition had no effect on their food choices (*β =* 0.49, *p* = 0.046 and *β =* 0.81, *p* < 0.000, respectively).

Trying to gain weight was associated with higher adherence to the Prudent (*β* = 0.25, *p* = 0.046), Western (*β =* 0.23, *p* = 0.042), and Alcohol patterns (*β =* 0.40, *p* = 0.002). Trying to lose weight was associated with greater adherence the Alcohol pattern (*β =* 0.26, *p* = 0.003) and lower adherence to the Western pattern (*β =* −0.20, *p* = 0.007). Being a current smoker was associated with higher adherence to the Alcohol pattern (*β =* 0.92, *p* < 0.001). MET minutes of physical activity were positively associated with the Prudent pattern score *(β =* 0.00, *p* = 0.004). Gender did not modify any of the observed associations (Supplementary Table S2). Hours of television watched and adherence to the alcohol pattern differed significantly for participants who identified as non-Hispanic white compared to those who identified as African American, Hispanic/Latino, Asian Pacific Islander, American Indian, Alaskan Native, or multi-racial (Supplementary Tables S2 and S3, *p* interaction = 0.0317). Associations between hours of television watched and adherence to the alcohol pattern were no longer significant in stratified models (Table 3).

## 4. Discussion

Findings from this study suggest that underlying lifestyle and behavioral factors are associated with adherence to dietary patterns among four-year university students. Specifically, participants who reported never eating out had lower adherence to the Western and Alcohol patterns (characterized by higher intakes of refined grains, processed meats, and/or alcoholic beverages) and higher adherence to the Prudent dietary pattern (characterized by higher intakes of plant-based foods) compared to those who reported eating out frequently. Trying to lose weight was associated with lower adherence to the Western pattern, and greater adherence to the Alcohol dietary pattern. Given that we previously demonstrated these dietary patterns are correlated with measures of disease risk in this population [24], it is important to consider both eating out behavior and weight loss intention in designing interventions to improve overall dietary quality among university students. Moreover, these findings point to a continued need to consider multi-level strategies that utilize policy and environmental approaches such as offering healthier choices as the default on restaurant menus [53,54,55,56,57,58] and individualized approaches such as patient care for students suffering from disordered eating [59].

We did not find any differences in the associations between diet-related behaviors and overall diet patterns by gender, despite a large body of literature demonstrating this disparity [46,51,52,60,61,62]. For example, studies have observed that university-age men were more likely than women to engage in unhealthy behaviors (e.g., poor dietary practices and not exercising) [51,61]. Moreover, there is some evidence that associations between diet-related behaviors and weight differ by gender. In one study, weight loss intentions were more likely to be associated with exercise behaviors in female, compared to male, university students; yet male university students were more likely to have stronger associations between exercise and drinking behaviors [52]. It is possible that we did not observe the same differences here because of the relatively small number of male students in our sample (only ~34%). Given the potential interactions between health behaviors, gender, and underlying social determinants such as norms and values [46,51,52,60,61,62]; it is also possible that we did not fully capture the complex relationship between all the possible determinants of dietary behaviors in university students in our models.

Significant differences in the associations between television viewing behavior and adherence to the alcohol dietary pattern were observed by race/ethnicity (*p* interaction = 0.0317). However, these associations only appeared significant when adjusting for all other behavioral factors. In simplified models stratified by race/ethnicity, television viewing behavior was not a significant predictor of adherence to the alcohol dietary pattern. There is a large body of literature that suggests that SES is a stronger determinant of dietary behaviors than race/ethnicity (data on SES was not collected here) [49]. Future research should consider the intersectionality of factors such as age, gender, SES, and race/ethnicity in evaluating differences in diet and diet-related behaviors [47,48,49,50].

Overall, our results are consistent with previous literature examining eating out behavior, weight loss intention, and diet quality in adolescent and adult populations. Frequently eating out at restaurants has been associated with overall diets that are higher in total energy, saturated fat, and added sugars and lower in quality in both children and adults [63,64]. In two studies examining university students in Minnesota, Pelletier et al. (2013) and Laska et al. (2015) found that fast food intake was negatively associated with fruit and vegetable intake, more energy from fat, and higher intakes of added sugar [29,65]; however, neither study evaluated associations specifically with overall diet patterns. 

Students who reported intending to lose weight (52.9% of the sample) had lower adherence to the Western and higher adherence to the Alcohol dietary patterns. A recent review of barriers and enablers of healthy eating among young adults reported that desire for weight management was associated with increased fruit consumption [66,67,68], suggesting that weight reduction efforts may be associated with healthier patterns. However, we did not observe a relationship between weight loss intention and the prudent dietary pattern in the present study. Several studies have considered relationships between alcohol consumption (especially binge drinking behavior) and eating patterns, body satisfaction, and weight loss intention [52,69,70]; findings from these studies suggest that alcohol abuse may be associated with disordered eating and/or lower diet quality. In one study, binge drinking was associated with poor diet, unhealthy weight control, body dissatisfaction, and sedentary behavior [62]. The high intakes of diet soda and coffee in the alcohol pattern has also been associated with dieting behaviors in young adults [52,71]. We did not measure participants’ motivation for weight loss, but it is possible that weight loss intention is a mediator of such motivations and should be considered in future studies.

Interestingly, intention to gain weight (reported by 11.8% of sample) was associated with greater adherence to all three dietary patterns. Studies evaluating adolescents with weight gain intentions have found positive associations with intakes of fat, French fries, and potato chips, and inverse associations with healthier foods like fruit and green salads [72,73]. Similarly, we found that intention to gain weight was associated with the Western pattern, characterized by foods like French fries and refined grains. However, we also observed positive associations between intention to gain weight and the Prudent and Alcohol dietary patterns. It is unclear whether these individuals were following these dietary patterns in order to gain weight, or if there were specific aspects of their diet that they felt were more important for their weight gain goals. Efforts to educate university students about healthy strategies for weight gain as well as weight loss may be necessary to ensure that students are engaging in healthy weight management behaviors [32,72,73,74].

In contrast with previous studies, we did not find consistent associations between the influence of peers, family, and/or nutrition across dietary patterns. In this study, we observed that *negative* or *strongly negative* family effects on food choice were inversely associated with adherence to the Western pattern. In contrast, previous research has found that youth perceptions of family members eating healthfully and/or supporting healthy eating has been associated with lower adherence to the “risky” or “fast food” dietary patterns characterized by snack and/or fast food intake [22,75] and higher adherence to the vegetable and fruit pattern [22,76]. Dickens and Ogden (2014) also observed that parents’ own dietary behaviors were stronger predictors of child dietary behaviors than parental control after the child left home [77]. It is possible that university students who viewed their family as negatively impacting their dietary behaviors were more motivated to change their diets after leaving home. Such students may have been eating healthier dietary patterns compared to their families but still engaged in dietary habits that were not in line with the Prudent pattern. Additional qualitative research could help elucidate the complex relationship between how family members continue to influence university students’ underlying motivations for dietary behaviors as they move away from home.

We also observed that participants who reported that nutrition had either a positive or negative influence on their food choices also had higher adherence to the Prudent pattern. There were no significant associations between the perceived effects of nutrition and the Western and Alcohol patterns. Previous research has shown that values around health and nutrition are associated with more healthful dietary behaviors among university students [41,78]. Here, students who identified nutrition as an influence on their food choices in any capacity (regardless of the direction) may have been more apt to make healthier decisions overall; these same individuals may also have higher levels of nutrition knowledge, which has been shown to be positively related to diet quality in other studies evaluating university-age populations [76,79]. Future research evaluating predictors of dietary patterns should consider possible interactive effects between factors like nutrition knowledge and perceptions around how/whether nutrition influences food choices.

Similarly, the effect of friends on food choice was only significantly associated with adherence to the Alcohol pattern, consistent with the increasing peer-pressure to drink that likely occurs during this life stage [80]. Yet, other studies have found that friends can also play a positive role in promoting fruit and vegetable intake during the university years, which was not observed here [22,76]. Unlike other research among US university students, we did not find significant associations between the perceived effects of culture/religion and dietary patterns or place of residence (living on vs. off campus) and dietary patterns [35,40,41,75,81].

Together, these findings indicate that nutrition interventions to improve diet quality among university students should include messaging and/or policies that encourage students to minimize eating out and to engage in healthy weight management behaviors. Several studies have shown that eating disorders are prevalent during the university years, with estimates ranging from 11 to 17% for university-age females and ~4% for males [46,59,82]—significantly higher than the estimated lifetime prevalence of eating disorders, which range from 0.3 to 3.9% [83]. Therefore, it is important to use language that encourages healthy weight-related behaviors across the weight-spectrum (underweight to obese) and does not incite unhealthy weight-related behaviors among individuals predisposed to disordered eating [35,84]. In designing these types of programs, initial qualitative research can help elucidate the types of weight-related messaging that resonates with the target population and does not impose unintended harm to those at risk of disordered eating.

There are some inherent limitations to this study. The cross-sectional design of our analysis does not allow us to infer causality, but only to evaluate associations between behavioral factors and dietary patterns. We used FFQ data to measure diet and the questionnaire from the Aerobics Research Aerobics Center Longitudinal Study [43,44,45] to measure physical activity via self-report, which are subject to recall and social desirability bias [85,86,87]. It is also possible that the behavioral factors evaluated here are indicators of other behavioral patterns [27,88,89,90] and/or that these associations change over time within individuals [30]. Moreover, given that dietary decisions are complex and multifaceted [2,3,4,5,6], there is likely residual confounding from behavioral/lifestyle factors that were not evaluated in this study or accounted for in our models. In particular, future research should account for SES (which was not collected here) and consider differences in both dietary determinants and diet quality based on gender, SES, and race/ethnicity, so as to further tailor interventions and policies to meet the needs of all populations [91,92,93]. This study was conducted using a motivated population of university freshmen attending a private, highly-ranked university in the Northeastern United States, mostly healthy non-Hispanic white females. Therefore, findings may not be generalizable to other university-age populations and do not capture any changes in dietary patterns or health behaviors that may occur over the university years. Moreover, since the sample of freshmen included in our analyses entered university between 1998 and 2007, our findings may not be generalizable to university students today.

Nevertheless, there are several notable strengths. First, our analytic sample was large (*n* = 630) even after omitting participants with missing data for all variables of interest. Second, this study employed an empirical approach to identifying overall dietary patterns, rather than focusing on individual nutrients or food groups. As dietary patterns are a more robust indicator of disease risk than individual nutrient or food intake [94], studies like this are needed to understand behavioral predictors of the total diet [24,95]. Finally, all behavioral correlates of interest were identified based in the existing understanding of behavioral predictors of diet.

## 5. Conclusions

Given the importance of the university years in establishing lifelong dietary habits [12,14], it is imperative to better understand potential drivers of dietary behavior in the university-age population. Our findings suggest that targeting dining out behavior and/or promoting healthy strategies for weight loss or weight gain may help improve the overall composition of the diets of university freshmen. Future studies should evaluate longitudinal trends in dietary patterns and underlying behavioral factors to determine whether these associations persist over time and/or whether new associations emerge.

Additionally, efforts to identify if the same behavioral factors are important determinants of diet in different university contexts would help inform future tailored intervention strategies aimed at reaching those most at risk of chronic disease. Future controlled interventions targeting both eating out and weight loss behaviors in university students with culturally sensitive messaging could also help elucidate whether these associations are causal, and the effectiveness of interventions targeted toward the aforementioned behavioral predictors. A better understanding of factors that influence university students’ dietary patterns can inform policies, programs, and interventions to improve diet quality, which has important implications for the overall reduction of chronic disease risk for current and future generations.

## Figures and Tables

**Table 1:** Overall sample characteristics of Freshman participants (*n* = 630).

Female (%)	65.9
Age, years	18.5 ± 0.5
Race/Ethnicity	
Non-Hispanic White (%)	75.4
African American (%)	3.2
Hispanic (%)	3.6
Other (%)	17.8
Live on campus (%)	100.0
Non-smoker (%)	97.5
Physically active ^a^ (%)	83.8
Adequate sleep ^b^ (%)	42.0
BMI (kg/m^2^) ^c^	22.8 ± 3.0
Intention to lose weight (%)	52.9
Intention to gain weight (%)	11.8
Watched less than 1 h of TV/day (%)	64.5
Food choice negatively or strongly negatively affected by:	
Friends (%)	21.4
Family (%)	9.5
Living situation (%)	55.2
Food availability/convenience (%)	60.9
Religion/culture (%)	5.4
Nutrition (%)	1.0
Cost (%)	30.6
Sometimes or often ate:	
In the dining hall (%)	99.7
Self-prepared meals (%)	14.4
Out at restaurants/take out ^d^ (%)	41.4

All values are mean ± SD unless otherwise noted. ^a^ Physically active defined as ≥500 metabolic equivalent of task (MET) minutes/week. ^b^ Defined by the participant agreeing or strongly agreeing that they usually sleep 7–8 h a night. ^c^ BMI was calculated from measured height and weight. ^d^ Referred to throughout as “eating out”.

**Table 2:** Associations between behavioral factors and dietary pattern scores (*n* = 630).

	Prudent Dietary Pattern			Western Dietary Pattern			Alcohol Dietary Pattern		
Predictors	*β*	*se*	*p*	*β*	*se*	*p*	*β*	*se*	*p*
Eating out									
Never	0.54	0.26	0.037 *	−0.91	0.23	<0.000 *	−0.85	0.26	0.001 *
Rarely	0.31	0.21	0.137	−0.69	0.18	<0.001 *	−0.54	0.21	0.009 *
Sometimes	0.15	0.21	0.467	−0.63	0.19	0.001 *	−0.32	0.21	0.118
Frequently	—	—	—	—	—	—	—	—	—
Friends effect food choice									
Strongly positive/Positive	−0.08	0.10	0.430	0.08	0.09	0.370	0.15	0.10	0.130
Strongly negative/Negative	0.00	0.11	0.979	−0.08	0.10	0.410	0.26	0.11	0.017 *
No effect	—	—	—	—	—	—	—	—	—
Family effects food choice									
Strongly positive/Positive	0.07	0.10	0.492	−0.01	0.09	0.940	0.02	0.10	0.872
Strongly negative/Negative	−0.06	0.16	0.684	−0.33	0.14	0.021 *	−0.19	0.16	0.224
No effect	—	—	—	—	—	—	—	—	—
Nutrition effects food choice									
Strongly positive/Positive	0.49	0.11	<0.000 *	−0.17	0.10	0.096	0.04	0.11	0.737
Strongly negative/Negative	0.81	0.40	0.046 *	0.42	0.36	0.244	−0.23	0.40	0.572
No effect	—	—	—	—	—	—	—	—	—
Hours per day of TV									
0 h	0.61	0.44	0.162	−0.39	0.39	0.325	(*p*-interaction = 0.0317) ^†^		
<1 h	0.52	0.44	0.237	−0.28	0.39	0.471			
1 h	0.43	0.44	0.329	−0.35	0.40	0.374			
2 h	0.42	0.45	0.357	−0.03	0.40	0.931			
3 h	0.17	0.47	0.720	−0.11	0.42	0.789			
4 h	0.63	0.62	0.311	−0.21	0.56	0.711			
5 or more hours	—	—	—	—	—	—			
Tried to lose weight									
Yes	0.14	0.08	0.103	−0.20	0.08	0.007 *	0.26	0.08	0.003 *
No	—	—	—	—	—	—	—	—	—
Tried to gain weight									
Yes	0.25	0.13	0.046 *	0.23	0.11	0.042 *	0.40	0.13	0.002 *
No	—	—	—	—	—	—	—	—	—
Physical Activity (MET minutes)	0.00	0.00	0.004 *	−0.00	0.00	0.637	0.00	0.00	0.095
Race									
Non-Hispanic Black	−0.30	0.22	0.190	0.52	0.20	0.011 *	−0.44	0.22	0.049 *
Hispanic, Latin American	−0.07	0.21	0.738	−0.18	0.19	0.332	0.10	0.21	0.643
Other	−0.19	0.10	0.066	−0.01	0.09	0.873	0.03	0.10	0.777
Non-Hispanic White	—	—	—	—	—	—	—	—	—
Gender									
Female	0.22	0.09	0.017 *	−0.59	0.08	<0.000 *	0.08	0.09	0.383
Male	—	—	—	—	—	—	—	—	—
Current smoker									
Yes	0.14	0.24	0.561	−0.13	0.22	0.556	0.92	0.25	<0.001 *
No	—	—	—	—	—	—	—	—	—
Age	0.13	0.07	0.064	−0.07	0.06	0.277	0.12	0.07	0.092

Models were run with the following predictors included: frequency of eating out, perceived influence of friends on food choice, perceived influence of family on food choice, perceived influence of nutrition on food choice, reported hours of television watched per day, attempt to gain weight in the last year, attempt to lose weight in the last year, metabolic equivalent of task (MET) minutes of physical activity, and current smoking status. All results are adjusted for age, race, and gender. Interactions by gender were not significant (Supplementary Table S2). Interactions by race that were significant are shown above and in Supplementary Table S3. ^†^ The interaction model included race re-categorized as non-Hispanic white and all other racial/ethnic groups (including African Americans, Hispanic/Latino, Asian Pacific Islander, American Indian, and Alaskan Native, and multi-racial), as less than 25% of our sample identified as other than non-Hispanic white. Models stratified by race are shown in Table 3. * *p* < 0.05. — reference category for all categorical variables.

**Table 3:** Associations between behavioral factors and alcohol dietary pattern scores by race/ethnicity.

	Alcohol Dietary Pattern					
	Non-Hispanic White (*n* = 475)			All Other Races/Ethnicities (*n* = 155)		
Predictors	*β*	*se*	*p*	*β*	*se*	*p*
Hours per day of TV						
0 h	−0.57	0.56	0.309	0.30	0.78	0.969
<1 h	−0.32	0.56	0.568	−0.29	0.78	0.708
1 h	−0.28	0.56	0.620	−0.50	0.79	0.528
2 h	−0.36	0.57	0.535	−0.12	0.81	0.884
3 h	−0.34	0.59	0.565	0.66	0.94	0.483
4 h	−1.23	0.87	0.160	−0.90	1.00	0.371
5 or more hours	—	—	—	—	—	—
Gender						
Female	0.04	0.09	0.686	0.07	0.19	0.734
Male	—	—	—	—	—	—
Age	0.11	0.08	0.189	0.08	0.15	0.573

The models were run with the following predictor included: reported hours of television watched per day. All results are adjusted for age and gender. Models were stratified by non-Hispanic white and all other racial/ethnic groups (including African Americans, Hispanic/Latino, Asian Pacific Islander, American Indian, and Alaskan Native, and multi-racial), as less than 25% of our sample identified as other than non-Hispanic white. None of the overall F-tests were significant at *p* < 0.05. — reference category for all categorical variables.

## Supplementary Materials

The following are available online at http://www.mdpi.com/2072-6643/10/6/716/s1, Supplementary Table S1: Bivariate associations between behavioral factors and dietary pattern scores (*n* = 630), Supplementary Table S2: Differences in associations between behavioral factors and dietary pattern scores by gender (*n* = 630), Supplementary Table S3: Differences in associations between behavioral factors and dietary pattern scores by race/ethnicity (*n* = 630).
