# Supplementary material for: Behavioral Correlates of Empirically-Derived Dietary Patterns among University Students
Source: Nutrients. 2018 Jun 3;10(6):716. doi: 10.3390/nu10060716 (PMC6024866; doi:10.3390/nu10060716)
Supplement: Supplementary file 1 [file nutrients-10-00716-s001.pdf]

Supplementary Table S1. Bivariate associations between behavioral factors and dietary pattern scores (n=630)

	Prudent Dietary Pattern		
**Individual Predictors**	*β*	F statistic	p-value
Ate at dining hall		0.9	0.4216
Never	1.04		
Rarely	-0.71		
Sometimes	-0.30		
Frequently	0.00		
Self-prepared meals		0.3	0.8625
Never	-0.04		
Rarely	0.03		
Sometimes	0.02		
Frequently	0.08		
Eating out[a]		6.7	0.0002*
Never	0.35		
Rarely	0.10		
Sometimes	-0.17		
Frequently	-0.44		
Friends influence food choice		0.6	0.5297
Strongly positive/Positive	-0.00		
Strongly negative/Negative	0.07		
No effect	-0.04		
Family effects food choice		0.8	0.4643
Strongly positive/Positive	0.03		
Strongly negative/Negative	-0.05		
No effect	-0.09		
Living situation effects food choice		1.4	0.2606
Strongly positive/Positive	0.10		
Strongly negative/Negative	-0.05		
No effect	-0.02		
Food availability effects food choice		1.8	0.1630
Strongly positive/Positive	0.11		
Strongly negative/Negative	-0.06		
No effect	0.00		
Nutrition effects food choice		16.4	<0.0001*
Strongly positive/Positive	0.09		
Strongly negative/Negative	0.31		

No effect	-0.53		
Culture/religion effects food choice		0.8	0.4315
Strongly positive/Positive	-0.02		
Strongly negative/Negative	0.21		
No effect	-0.02		
Cost effects food choice		0.1	0.9395
Strongly positive/Positive	-0.04		
Strongly negative/Negative	0.01		
No effect	-0.00		
Hours of TV watched[b]		3.0	0.0068*
0 hours	0.18		
<1 hour	0.02		
1 hour	-0.14		
2 hours	-0.17		
3 hours	0.40		
4 hours	-0.16		
5 or more hours	-0.67		
Adequate sleep[c]		4.2	0.0419*
Yes	0.09		
No	-0.08		
Tried to lose weight		7.0	0.0083*
Yes	0.09		
No	-0.12		
Tried to gain weight		0.7	0.3884
Yes	0.09		
No	-0.02		
Perceived life control[d]		1.8	0.1657
Not in control at all	-0.25		
Completely in control	0.05		
Neutral	-0.09		
Physical activity (MET minutes)	0.00	10.3	0.0014*
Current smoker		0.1	0.7272
Yes	0.08		
No	-0.01		

Individual Predictors	Western Dietary Pattern		
	β	F statistic	p-value
Ate at dining hall		1.6	0.1892
Never	0.29		
Rarely	-0.50		
Sometimes	-0.45		
Frequently	-0.02		
Self-prepared meals		1.7	0.1642
Never	0.01		
Rarely	-0.04		
Sometimes	-0.17		
Frequently	-0.63		
Eating out[a]		8.5	<0.0001*
Never	-0.42		
Rarely	-0.10		
Sometimes	0.05		
Frequently	0.72		
Friends influence food choice		6.6	0.0015*
Strongly positive/Positive	-0.03		
Strongly negative/Negative	-0.28		
No effect	0.08		
Family effects food choice		7.7	0.0005*
Strongly positive/Positive	0.02		
Strongly negative/Negative	-0.49		
No effect	0.01		
Living situation effects food choice		0.4	0.6682
Strongly positive/Positive	-0.03		
Strongly negative/Negative	-0.01		
No effect	-0.10		
Food availability effects food choice		0.7	0.5076
Strongly positive/Positive	-0.04		
Strongly negative/Negative	-0.01		
No effect	-0.16		
Culture/religion effects food choice		1.9	0.1517
Strongly positive/Positive	0.03		
Strongly negative/Negative	-0.34		
No effect	-0.02		

Nutrition effects food choice		7.6	0.0005*
Strongly positive/Positive	-0.09		
Strongly negative/Negative	0.59		
No effect	0.28		
Cost effects food choice		0.5	0.5804
Strongly positive/Positive	0.02		
Strongly negative/Negative	0.01		
No effect	-0.07		
Hours of TV watched[b]		3.8	0.0011*
0 hours	-0.21		
<1 hour	-0.02		
1 hour	-0.05		
2 hours	0.34		
3 hours	0.39		
4 hours	0.27		
5 or more hours	-0.04		
Adequate sleep[c]		0.1	0.8168
Yes	-0.02		
No	-0.04		
Tried to lose weight		42.9	<0.0001*
Yes	-0.26		
No	0.23		
Tried to gain weight		26.6	<0.0001*
Yes	0.50		
No	-0.10		
Perceived life control[d]		1.7	0.1938
Not in control at all	-0.53		
Completely in control	0.00		
Neutral	-007		
Physical activity (MET minutes)	0.00	0.5	0.4978
Current smoker		0.0	0.9492
Yes	-0.05		
No	-0.03		

	**Alcohol Dietary Pattern**		
**Individual Predictors**	β	F statistic	p-value
Ate at dining hall		0.2	0.9951

Never	-0.16		
Rarely	-0.15		
Sometimes	0.04		
Frequently	0.03		
Self-prepared meals		0.7	0.5745
Never	0.03		
Rarely	0.01		
Sometimes	0.17		
Frequently	0.24		
Eating out[a]		4.9	0.0022*
Never	-0.37		
Rarely	-0.04		
Sometimes	0.16		
Frequently	0.37		
Friends influence food choice		4.1	0.0168*
Strongly positive/Positive	0.09		
Strongly negative/Negative	0.20		
No effect	-0.08		
Family effects food choice		1.4	0.2585
Strongly positive/Positive	0.08		
Strongly negative/Negative	-0.09		
No effect	-0.05		
Living situation effects food choice		1.0	0.3378
Strongly positive/Positive	-0.04		
Strongly negative/Negative	0.05		
No effect	0.12		
Food availability effects food choice		0.9	0.3980
Strongly positive/Positive	0.03		
Strongly negative/Negative	0.01		
No effect	0.19		
Culture/religion effects food choice		0.7	0.5195
Strongly positive/Positive	-0.07		
Strongly negative/Negative	-0.06		
No effect	0.05		
Nutrition effects food choice		0.1	0.9168
Strongly positive/Positive	0.04		
Strongly negative/Negative	-0.08		
No effect	0.00		

Cost effects food choice		0.2	0.8299
Strongly positive/Positive	-0.01		
Strongly negative/Negative	0.06		
No effect	0.03		
Hours of TV watched[b]		0.9	0.4839
0 hours	-0.04		
<1 hour	0.08		
1 hour	0.03		
2 hours	0.08		
3 hours	0.20		
4 hours	-0.69		
5 or more hours	0.33		
Adequate sleep[c]		1.4	0.2383
Yes	-0.02		
No	0.07		
Tried to lose weight		11.0	0.0010*
Yes	0.15		
No	-0.10		
Tried to gain weight		7.8	0.0054*
Yes	0.33		
No	-0.01		
Perceived life control[d]		0.9	0.4212
Not in control at all	-0.03		
Completely in control	-0.00		
Neutral	0.11		
Physical activity (MET minutes)	0.00	1.6	0.2068
Current smoker		15.2	0.0001*
Yes	0.97		
No	0.01		

[a] Participants could indicate how they obtained most of their meals in the past academic year: in the dining hall; prepared in their dorm, apartment, or home; or out at restaurants (never, rarely, sometimes, often).
[b]Participants could indicate whether they watched: 0 hours, less than 1 hour, 2 hours, 3 hours, 4 hours, or 5 or more hours of television per day.
[c]Adequate sleep was defined by the participant indicating that they agree or strongly agree that they usually sleep between 7 and 8 hours a night.
[d]Participants indicated on a scale of 1-10 their sense of control over their own life: 1 being not in control at all and 10 being in complete control. These responses were further categorized as such: not in control at all (respondent indicated 1-3 on the scale), in complete control (respondent indicated 8-10 on the scale), neutral (respondent indicated 4-7 on the scale).

Supplementary Table S2. Differences in associations between behavioral factors and dietary pattern scores by gender (n=630)

Predictors	Prudent Dietary Pattern			Western Dietary Pattern			Alcohol Dietary Pattern		
	β	*se*	*p*	*β*	*se*	*p*	*β*	*se*	*p*
Eating out									
Never Female*	-0.98	0.58	0.090	0.01	0.51	0.982	-0.22	0.57	0.701
*Never*Male*	—	—	—	—	—	—	—	—	—
*Rarely*Female*	-0.40	0.42	0.344	-0.22	0.37	0.562	-0.77	0.42	0.067
Rarely Male*	—	—	—	—	—	—	—	—	—
Sometimes Female*	-0.23	0.43	0.583	0.08	0.38	0.842	-0.54	0.42	0.205
Sometimes Male*	—	—	—	—	—	—	—	—	—
Frequently Female*	—	—	—	—	—	—	—	—	—
*Frequently*Male*	—	—	—	—	—	—	—	—	—
Friends effect food choice									
*Strongly positive/Positive*Female*	-0.15	0.21	0.472	-0.02	0.18	0.913	0.02	0.21	0.922
*Strongly positive/Positive*Male*	—	—	—	—	—	—	—	—	—
Strongly negative/Negative Female*	0.15	0.26	0.569	0.43	0.23	0.064	-0.16	0.26	0.542
Strongly negative/Negative Male*	—	—	—	—	—	—	—	—	—
No effect Female*	—	—	—	—	—	—	—	—	—
No effect Male*	—	—	—	—	—	—	—	—	—
Family effects food choice									
*Strongly positive/Positive*Female*	0.10	0.22	0.646	-0.20	0.19	0.291	0.05	0.21	0.820
*Strongly positive/Positive*Male*	—	—	—	—	—	—	—	—	—
Strongly negative/Negative Female*	-0.01	0.39	0.976	0.19	0.35	0.587	0.24	0.39	0.535
Strongly negative/Negative Male*	—	—	—	—	—	—	—	—	—
No effect Female*	—	—	—	—	—	—	—	—	—
No effect Male*	—	—	—	—	—	—	—	—	—
Nutrition effects food choice									
*Strongly positive/Positive*Female*	0.08	0.23	0.725	-0.05	0.20	0.791	-0.27	0.23	0.228
*Strongly positive/Positive*Male*	—	—	—	—	—	—	—	—	—
Strongly negative/Negative Female*	-0.28	0.89	0.754	0.69	0.79	0.379	-0.44	0.88	0.614
Strongly negative/Negative Male*	—	—	—	—	—	—	—	—	—
No effect Female*	—	—	—	—	—	—	—	—	—
No effect Male*	—	—	—	—	—	—	—	—	—
Hours per day of TV									
*0 hours*Female*	-0.64	1.12	0.566	-0.49	0.99	0.624	1.71	1.11	0.124

*0 hours*Male*	—	—	—	—	—	—	—	—	—
*<1 hour*Female*	-0.39	1.11	0.725	-0.39	0.99	0.696	1.5	1.1	0.17
*<1 hour*Male*	—	—	—	—	—	—	—	—	—
*1 hour*Female*	-0.70	1.11	0.532	-0.696	0.99	0.484	1.50	1.10	0.175
Hours per day of TV (cont.)									
*1 hour*Male*	—	—	—	—	—	—	—	—	—
*2 hours*Female*	-0.28	1.13	0.806	-0.85	1.01	0.400	1.70	1.13	0.131
*2 hours*Male*	—	—	—	—	—	—	—	—	—
*3 hours*Female*	-0.46	1.16	0.695	-1.14	1.03	0.271	0.72	1.15	0.533
*3 hours*Male*	—	—	—	—	—	—	—	—	—
*4 hours*Female*	—	—	—	—	—	—	—	—	—
*4 hours*Male*	—	—	—	—	—	—	—	—	—
*5 or more hours*Female*	—	—	—	—	—	—	—	—	—
*5 or more hours*Male*	—	—	—	—	—	—	—	—	—
Tried to lose weight									
*Yes*Female*	0.17	0.18	0.359	0.17	0.16	0.285	0.01	0.18	0.935
*Yes*Male*	—	—	—	—	—	—	—	—	—
*No*Female*	—	—	—	—	—	—	—	—	—
*No*Male*	—	—	—	—	—	—	—	—	—
Tried to gain weight									
*Yes*Female*	-0.50	0.28	0.083	0.20	0.25	0.427	-0.48	0.28	0.091
*Yes*Male*	—	—	—	—	—	—	—	—	—
*No*Female*	—	—	—	—	—	—	—	—	—
*No*Male*	—	—	—	—	—	—	—	—	—
Physical Activity (MET minutes)*Female	0.00	0.00	0.424	0.00	0.00	0.665	0.00	0.00	0.505
Physical Activity (MET minutes)*Male	—	—	—	—	—	—	—	—	—
Current smoker									
*Yes*Female*	0.51	0.53	0.336	-0.33	0.47	0.479	0.28	0.52	0.598
*Yes*Male*	—	—	—	—	—	—	—	—	—
*No*Female*	—	—	—	—	—	—	—	—	—
*No*Male*	—	—	—	—	—	—	—	—	—

Models were run with the following predictors included: frequency of eating out, perceived influence of friends on food choice, perceived influence of family on food choice, perceived influence of nutrition on food choice, reported hours of television watched per day, attempt to gain weight in the last year, attempt to lose weight in the last year, metabolic equivalent of task (MET) minutes of physical activity, and current smoking status. Interactions between each of the aforementioned behaviors and gender were also tested (shown above). All results are adjusted for age and race.

None of the overall F-tests were significant at $p<0.05$.

— reference category for all categorical variables

Supplementary Table S3. Differences in associations between behavioral factors and dietary pattern scores by race/ethnicity (n=630)

	Prudent Dietary Pattern			Western Dietary Pattern			Alcohol Dietary Pattern		
**Predictors**	*β*	*se*	*p*	*β*	*se*	*p*	*β*	*se*	*p*
Eating out									
Never Non-Hispanic White*	0.49	0.60	0.410	0.24	0.53	0.653	0.19	0.59	0.743
*Never*Male*	—	—	—	—	—	—	—	—	—
*Rarely*Non-Hispanic White*	0.13	0.43	0.762	0.52	0.38	0.174	0.08	0.42	0.856
Rarely All Other Races/Ethnicities*	—	—	—	—	—	—	—	—	—
Sometimes Non-Hispanic White*	-0.09	0.43	0.844	0.31	0.39	0.419	0.27	0.43	0.535
Sometimes All Other Races/Ethnicities*	—	—	—	—	—	—	—	—	—
Frequently Non-Hispanic White*	—	—	—	—	—	—	—	—	—
*Frequently*All Other Races/Ethnicities*	—	—	—	—	—	—	—	—	—
Friends effect food choice									
*Strongly positive/Positive*Non-Hispanic Whit*	-0.03	0.22	0.901	-0.35	0.20	0.076	-0.16	0.22	0.460
*Strongly positive/Positive*All Other Races/Ethnicities*	—	—	—	—	—	—	—	—	—
*Strongly negative/Negative*Non-Hispanic White*	-0.06	0.28	0.842	-0.17	0.25	0.483	-0.15	0.27	0.590
*Strongly negative/Negative*All Other Races/Ethnicities*	—	—	—	—	—	—	—	—	—
No effect Non-Hispanic White*	—	—	—	—	—	—	—	—	—
No effect All Other Races/Ethnicities*	—	—	—	—	—	—	—	—	—
Family effects food choice									
*Strongly positive/Positive*Non-Hispanic White*	0.16	0.24	0.504	-0.13	0.22	0.554	-0.10	0.24	0.680
*Strongly positive/Positive*All Other Races/Ethnicities*	—	—	—	—	—	—	—	—	—
Strongly negative/Negative Non-Hispanic White*	0.58	0.37	0.114	0.12	0.33	0.724	0.21	0.36	0.559
Strongly negative/Negative All Other Races/Ethnicities*	—	—	—	—	—	—	—	—	—
No effect Non-Hispanic White*	—	—	—	—	—	—	—	—	—
No effect All Other Races/Ethnicities*	—	—	—	—	—	—	—	—	—
Nutrition effects food choice									

*Strongly positive/Positive*Non-Hispanic White*	-0.46	0.25	0.067	-0.04	0.22	0.843	0.35	0.25	0.159
*Strongly positive/Positive*All Other Races/Ethnicities*	—	—	—	—	—	—	—	—	—
Nutrition effects food choice (cont.)									
Strongly negative/Negative Non-Hispanic White*	0.77	0.88	0.379	-0.98	0.78	0.210	-0.38	0.86	0.659
*Strongly negative/Negative*All Other Races/Ethnicities*	—	—	—	—	—	—	—	—	—
No effect Non-Hispanic White*	—	—	—	—	—	—	—	—	—
No effect All Other Races/Ethnicities*	—	—	—	—	—	—	—	—	—
Hours per day of TV									
*0 hours*Non-Hispanic White*	0.41	0.93	0.661	0.96	0.83	0.248	-0.26	0.91	0.776†
*0 hours*All Other Races/Ethnicities*	—	—	—	—	—	—	—	—	—
*<1 hour*Non-Hispanic White*	0.19	0.93	0.835	1.13	0.83	0.171	0.30	0.91	0.738†
*<1 hour*All Other Races/Ethnicities*	—	—	—	—	—	—	—	—	—
*1 hour*Non-Hispanic White*	0.32	0.93	0.727	1.23	0.83	0.147	0.58	0.91	0.527†
*1 hour*All Other Races/Ethnicities*	—	—	—	—	—	—	—	—	—
*2 hours*Non-Hispanic White*	0.38	0.95	0.687	0.97	0.85	0.256	0.32	0.94	0.735†
*2 hours*All Other Races/Ethnicities*	—	—	—	—	—	—	—	—	—
*3 hours*Non-Hispanic White*	-0.03	1.05	0.979	0.61	0.94	0.517	-0.74	1.04	0.477†
*3 hours*All Other Races/Ethnicities*	—	—	—	—	—	—	—	—	—
*4 hours*Non-Hispanic White*	0.65	1.29	0.618	1.90	1.15	0.100	-0.08	1.27	0.949†
*4 hours*All Other Races/Ethnicities*	—	—	—	—	—	—	—	—	—
*5 or more hours*Non-Hispanic White*	—	—	—	—	—	—	—	—	—
*5 or more hours*All Other Races/Ethnicities*	—	—	—	—	—	—	—	—	—
Tried to lose weight									
*Yes*Non-Hispanic White*	0.07	0.20	0.729	-0.01	0.18	0.977	0.32	0.20	0.105
*Yes*All Other Races/Ethnicities*	—	—	—	—	—	—	—	—	—
*No*Non-Hispanic White*	—	—	—	—	—	—	—	—	—
*No*All Other Races/Ethnicities*	—	—	—	—	—	—	—	—	—
Tried to gain weight									
*Yes*Non-Hispanic White*	0.13	0.28	0.653	-0.08	0.25	0.758	-0.10	0.28	0.713
*Yes*All Other Races/Ethnicities*	—	—	—	—	—	—	—	—	—
*No*Non-Hispanic White*	—	—	—	—	—	—	—	—	—
*No*All Other Races/Ethnicities*	—	—	—	—	—	—	—	—	—
Physical Activity (MET minutes)*Non-Hispanic White	-0.00	0.00	0.686	0.00	0.00	0.249	-0.00	0.00	0.322

Physical Activity (MET minutes)*All Other Races/Ethnicities	—	—	—	—	—	—	—	—	—
Current smoker									
*Yes*Non-Hispanic White*	-0.55	0.78	0.477	1.12	0.69	0.107	-1.50	0.768	0.052
*Yes*All Other Races/Ethnicities*	—	—	—	—	—	—	—	—	—
*No*Non-Hispanic White*	—	—	—	—	—	—	—	—	—
*No*All Other Races/Ethnicities*	—	—	—	—	—	—	—	—	—

Models were run with the following predictors included: frequency of eating out, perceived influence of friends on food choice, perceived influence of family on food choice, perceived influence of nutrition on food choice, reported hours of television watched per day, attempt to gain weight in the last year, attempt to lose weight in the last year, metabolic equivalent of task (MET) minutes of physical activity, and current smoking status. Interactions between each of the aforementioned behaviors and race/ethnicity were also tested (shown above). All results are adjusted for age and gender.

†overall F-test $p<0.05$ for the model evaluating associations between total hours of television watched per day and adherence to the alcohol pattern by race/ethnicity.

— reference category for all categorical variables
